# Thiolated chitosan/alginate/Emodin nanoparticles alleviate chronic kidney disease via gut microbiota regulation and intestinal barrier protection

**DOI:** 10.1128/spectrum.02360-25

**Published:** 2026-03-31

**Authors:** Wenbo Liu, Yuanyuan Zhang, Shuangchun Gu, La Zhang, Yuchi Wu, Lihua Huang, Fuhua Lu, Xusheng Liu, Zhaoyu Lu

**Affiliations:** 1 State Key Laboratory of Dampness Syndrome of Chinese Medicine, The Second Clinical College of Guangzhou University of Chinese Medicine, Guangzhou, China; 2 Nephrology Department, The Second Affiliated Hospital of Guangzhou University of Chinese Medicine, Guangzhou, China; Cleveland Clinic Lerner Research Institute, Cleveland, Ohio, USA

**Keywords:** chronic kidney disease, gut microbiota, intestinal barrier function, Emodin, thiolated chitosan-sodium alginate nanoparticles

## Abstract

Chronic kidney disease (CKD) is closely associated with gut microbiota dysbiosis and impaired intestinal barrier function, yet effective therapeutic strategies targeting the gut–kidney axis remain limited. This study aimed to develop a thiolated chitosan/sodium alginate-based nanoparticle system encapsulating emodin (TCS/ALG/Emodin-NPs), and to evaluate its therapeutic efficacy in CKD through modulation of gut microbiota and intestinal barrier protection. The nanoparticles were synthesized, characterized, and orally administered to CKD mice at varying doses, with irbesartan as a positive control. Our results showed that TCS/ALG/Emodin-NPs significantly improved renal function, reduced renal fibrosis, and restored gut microbiota balance, particularly increasing beneficial bacteria such as *Lactobacillus*. They also enhanced intestinal tight junction integrity and suppressed systemic inflammation. In a hydrogen peroxide-induced Caco-2 cell model, Emodin-NPs more effectively attenuated oxidative stress and apoptosis compared to free Emodin. The findings suggest that TCS/ALG/Emodin-NPs are a promising strategy for CKD treatment by reshaping gut microbiota and enhancing intestinal function.

**IMPORTANCE:**

While the gut–kidney axis has emerged as a critical target in chronic kidney disease (CKD) management, therapeutic strategies leveraging this pathway remain limited. This study demonstrates that colon-targeted TCS/ALG/Emodin-NPs not only enhance drug bioavailability but also exert dual therapeutic effects by rectifying gut dysbiosis and reinforcing intestinal barrier function—key mechanisms implicated in CKD progression. The superior efficacy of TCS/ALG/Emodin-NPs over free Emodin underscores the transformative potential of nanoparticle delivery systems in overcoming the pharmacokinetic limitations of herbal medicines. This research opens new avenues for the development of microbiota-targeted, anti-inflammatory therapies that can complement existing CKD treatments and potentially slow disease progression in affected patients.

## INTRODUCTION

Chronic kidney disease (CKD) has emerged as a significant global public health concern. The rising prevalence of risk factors such as diabetes, hypertension, and an aging population has led to a steady increase in CKD cases (1, 2), which places a substantial burden on healthcare systems and affects patients’ quality of life (3, 4). Consequently, the global nephrology community is intensifying efforts to discover more effective strategies to slow CKD progression.

One promising area of research is the gut–kidney axis, which highlights the intricate connection between gut microbiota and kidney function. In CKD patients, imbalances in gut microbial composition, known as dysbiosis, lead to increased production of harmful metabolites such as uremic toxins. Because kidney clearance is impaired, these uremic toxins accumulate in the body, exacerbating oxidative stress, chronic inflammation, and renal fibrosis, all of which accelerate renal deterioration (5, 6).

In addition, dysbiosis associated with CKD compromises the integrity of the intestinal barrier, facilitating the translocation of endotoxins such as lipopolysaccharides (LPS) into systemic circulation. This leakage triggers widespread inflammation, further damaging renal tissues (6, 7). Simultaneously, a decline in beneficial short-chain fatty acids (SCFAs), particularly butyrate, weakens gut health and amplifies inflammatory processes, forming a self-perpetuating cycle that accelerates CKD progression (8).

Targeting the gut microbiota and intestinal barrier thus represents a novel therapeutic avenue for CKD. In traditional Chinese medicine (TCM), rhubarb is widely used to treat CKD for its detoxifying and intestinal-cleansing properties. Contemporary research supports its therapeutic potential, especially that of its active compound, Emodin (Em), which has demonstrated the ability to reduce uremic toxins, regulate gut microbiota, and alleviate kidney injury (9–11).

However, the key active ingredients of rhubarb, such as Emodin, have poor water solubility and low oral utilization. The oral utilization rate of Emodin is only 3% (12, 13). Oral administration of rhubarb exhibits limited targeting efficiency toward the colon. While rectal administration of rhubarb extracts enhances colon-specific delivery, it suffers from short retention, poor mucosal adhesion, frequent dosing requirements, and low patient compliance, limiting its long-term efficacy (13, 14). These challenges underscore the need for more efficient delivery systems that enhance bioavailability and target Emodin to the colon.

Oral colon-specific drug delivery systems (OCDDS) have emerged as an innovative approach to address these limitations. These nano-formulations can enhance the solubility and bioavailability of the active ingredients in traditional Chinese medicine (15). More importantly, they were designed to release active compounds directly in the colon, thereby interacting more effectively with colonic microbiota and epithelial cells and providing sustained therapeutic effects (16). Among the various materials used for colon-targeted delivery, chitosan (CS) is particularly notable for its strong mucoadhesive properties due to its ability to form hydrogen bonds and electrostatic interactions with mucosal proteins (17). When thiolated, its adhesion is further enhanced (18, 19). Sodium alginate also shows promise, with excellent gel-forming ability and bioadhesion (20), making it another favorable candidate for OCDDS.

There remains a gap in the development of colon-targeted nano-delivery systems specifically designed to enhance Emodin’s therapeutic potential by acting on both the gut microbiota and the intestinal barrier in the context of CKD. To address this gap, we designed and evaluated a thiolated chitosan/sodium alginate-based nanoparticle system encapsulating Emodin (TCS/ALG/Emodin-NPs). This study aimed to improve Emodin’s bioavailability and colon-targeting capacity, assess its impact on gut microbiota composition and intestinal barrier function in CKD, and determine whether such modulation could translate into renal protection and reduced systemic inflammation.

## MATERIALS AND METHODS

### Preparation of thiochitosan/sodium alginate Emodin nanoparticles (TCS/ALG/Emodin-NPs)

To prepare thiochitosan, dissolve 5 g of CS (Sigma-Aldrich, Merck KGaA, Darmstadt, Germany) in 460 mL of distilled water under continuous stirring. Gradually add 3.486 g of HOBT (Sigma-Aldrich) until the solution becomes clear. Introduce 8.42 g of N-acetylcysteine (Sigma-Aldrich) and 19.785 g of EDC (Macklin, Shanghai, China) into the mixture. Adjust the pH to 5 using hydrochloric acid, and allow the reaction to proceed at room temperature for 3 hours. The resulting solution is then dialyzed using a 14,000 Da molecular weight cutoff membrane in deionized water for 3 days in the dark. After dialysis, the product is freeze-dried and stored at 4°C. Dissolve 30 mg of sodium alginate (ALG, Sigma-Aldrich) in 10 mL of deionized water. Slowly introduce 1.5 mL of calcium chloride solution (2 mg/mL) while stirring for 10 minutes. Separately, dissolve 6 mg of TCS in 2 mL of 1% acetic acid, and add it dropwise to the alginate mixture. Continue stirring for another 30 minutes. Following this, centrifuge the suspension at 1,200 rpm; discard the supernatant; resuspend the pellet in water; and freeze-dry to obtain the TCS/ALG nanoparticles. To fabricate the drug-loaded formulation, dissolve 30 mg of sodium alginate in 10 mL of deionized water and add 1.5 mL of calcium chloride solution (2 mg/mL) under gentle stirring for 10 minutes. Dissolve Emodin (Macklin) in 2 mL of ethanol and incorporate it into the alginate solution, stirring for another 10 minutes. Prepare 6 mg of TCS in 2 mL of 1% acetic acid, and add this to the Emodin-containing mixture dropwise. After an additional 30 minutes of stirring, centrifuge at 1,200 rpm to remove the supernatant. Resuspend the pellet in water and freeze-dry to obtain TCS/ALG/Emodin-NPs.

### Characterization of TCS/ALG/Emodin-NPs

Dissolve 3–5 mg of each sample in deuterium oxide and transfer to clean NMR tubes. Spectra are acquired at room temperature using an NMR spectrometer and analyzed with MestReNova software. Place a drop of the blank and TCS/ALG/Emodin-NP suspensions onto copper grids. After drying, examine the morphology using transmission electron microscopy (TEM). Prepare 1 mg/mL suspensions of the TCS/ALG/Emodin-NP samples. Load 1 mL of each into the measurement and determine particle size distribution via dynamic light scattering. Mix each sample with potassium bromide; press into pellets; and analyze using Fourier-transform infrared (FTIR) in the 500–4,000 cm⁻¹ wavelength range.

### Drug release study

Seal 0.1 mL of the TCS/ALG/Emodin-NP solution in a dialysis bag and place it in a 5 mL centrifuge tube containing 3 mL of phosphate-buffered saline (PBS) buffer with 5% Tween 80. Conduct the release test at 1,500 rpm. Experiments are carried out in buffers of pH 1.2, 6.8, and 7.4. At defined intervals, the entire dialysis medium is withdrawn for analysis and replaced with fresh PBS. Drug release is monitored over 72 hours using a UV–Vis spectrophotometer.

### Experimental animals and reagents

Seventy specific pathogen-free (SPF) male C57BL/6 mice (weighing 20–22 g) were obtained from Guangdong GemPharmatech Co. Ltd (no. 44824700036115). The animals were housed in the SPF Animal Experimental Center of Guangdong Provincial Hospital of Chinese Medicine (animal experiment license: SYXK-2023-0094), under controlled conditions: a 12-hour light/dark cycle, ambient temperature of (20 ± 2)°C, and relative humidity of (50 ± 5)%. Mice were allowed *ad libitum* access to standard chow and water. Following a 1-week acclimatization period, experimental procedures were initiated.

### CKD modeling and Emodin-NP intervention

To minimize disruption to the peritoneal and gut microbiota typically caused by 5/6 nephrectomy, we opted to induce CKD using a 0.2% adenine-enriched diet, as previously described (21). The diet was provided by the Guangdong Provincial Medical Experimental Animal Center. After a 1-week acclimatization period, C57BL/6 mice were randomly divided into seven groups (*n* = 10 per group): (i) normal control; (ii) adenine only (Ade); (iii) Ade + low-dose Emodin (Emodin-L, 20 mg/kg); (iv) Ade + high-dose Emodin (Emodin-H, 40 mg/kg); (v) Ade + low-dose TCS/ALG/Emodin-NPs (Emodin-NPs-L 20 mg/kg); (vi) Ade + high-dose TCS/ALG/Emodin-NPs (Emodin-NPs-H, 40 mg/kg); and (vii) Ade + irbesartan (40 mg/kg).

The Emodin dosages of 20 and 40 mg/kg used in this study were selected based on previous pharmacological studies demonstrating their efficacy and safety in murine models of kidney disease. Prior research has shown that Emodin at these concentrations exhibits significant anti-inflammatory and antifibrotic effects without causing notable toxicity in mice (9, 22). Moreover, our preliminary experiments confirmed that these dosages were well tolerated. Therefore, both 20 and 40 mg/kg were employed to evaluate potential dose-dependent effects in this study. Numerous clinical studies have established irbesartan as an effective therapeutic agent for CKD, demonstrating significant efficacy in delaying disease progression and improving renal function, and the experimental dose in mice is approximately 40 mg/kg (23, 24). Therefore, we chose irbesartan 40 mg/kg as the positive control drug for this study. Renal fibrosis induced by adenine progresses notably over a 4- to 6-week period, encompassing both the early injury phase (weeks 1–2) and the stable fibrosis stage (weeks 4–6) (25). Accordingly, we chose a 6-week treatment window to evaluate the therapeutic impact.

So in this study, mice in all treatment groups, except for the normal group, received the 0.2% adenine diet continuously for 6 weeks. Mice in the Ade + Emodin groups were administered Emodin at either 20 mg/kg/day (Emodin-L) or 40 mg/kg/day (Emodin-H); mice in the Ade + Emodin nanoparticle groups were administered TCS/ALG/Emodin-NPs at either 20 mg/kg/day (Emodin-NPs-L) or 40 mg/kg/day (Emodin-NPs-H), while the Ade + irbesartan group received irbesartan at 40 mg/kg/day (Sanofi Winthrop Industries, Inc.) via intragastric gavage. The Ade and normal control groups received an equivalent volume of distilled water daily. Body weight and food intake were monitored weekly throughout the study.

### Histological analysis

Following euthanasia under deep anesthesia, kidney tissues were collected and fixed in 4% paraformaldehyde. Samples were then embedded in paraffin, sectioned at a thickness of 3 μm, and stained using hematoxylin and eosin (H&E) as well as Masson’s trichrome staining. Renal parenchymal abnormalities were identified by the presence of one or more of the following: tubular inflammation, dilation or loss, atrophy, interstitial inflammation, or fibrosis. Quantitative evaluation was performed by visually estimating the proportion of abnormal and fibrotic regions in well-oriented kidney sections encompassing both cortex and medulla, expressed as a percentage of the total tissue area (21).

### Measurement of serum creatinine and urea nitrogen

Levels of serum creatinine (Scr), urea nitrogen, and albumin were determined using commercial assay kits (Nanjing Jiancheng Bioengineering Institute, Nanjing, China) for creatinine and blood urea nitrogen (BUN), following the manufacturer’s protocols.

### 16S rRNA sequencing and data analysis

Fecal DNA was extracted using the NucleoSpin Soil kit (Macherey-Nagel, Germany). PCR was performed with 30 ng of high-quality genomic DNA and fusion primers under optimized thermal cycling conditions. PCR products were purified using Agencourt AMPure XP magnetic beads and eluted in elution buffer. Libraries were constructed by adding index labels, followed by quality assessment of fragment size and concentration using the Agilent 2100 Bioanalyzer. Sequencing was conducted on the MGI2000 platform.

Raw reads were filtered to obtain high-quality clean data for downstream analyses. Sequence reads were merged into tags based on overlapping regions using FLASH software (v.1.2.11). Operational taxonomic units (OTUs) were generated by clustering tags, which were then annotated against reference databases. Further analyses included species composition assessment, intergroup diversity comparison, and correlation analysis based on OTU and taxonomic annotations.

### Enzyme-linked immunosorbent assay

The concentrations of diamine oxidase (DAO), LPS, interleukin-1β (IL-1β), and IL-6 in serum were determined using commercially available enzyme-linked immunosorbent assay kits, according to the manufacturers’ instructions. Kits for mouse IL-1β and IL-6 were obtained from Absin (Shanghai, China), while DAO and LPS assays were purchased from Cusabio (Wuhan, China).

### Cell culture and treatment

Human colorectal cells (Caco-2; ATCC, Manassas, VA, USA) were maintained at 37°C in a humidified incubator with 5% CO_2_. The cells were grown in Minimum Essential Medium (Gibco, Grand Island, NY, USA), supplemented with 20% fetal bovine serum (Gibco), 100 U/mL penicillin, and 100 μg/mL streptomycin (Gibco).

To simulate the intestinal mucosal oxidative stress injury caused by uremic toxins in CKD, hydrogen peroxide (H_2_O_2_) induced Caco-2 cell injury was carried. Caco-2 cells were exposed to 100 μmol/L hydrogen peroxide (H_2_O_2_; Boster, China) for 24 hours. For treatment groups, Emodin or Emodin-NPs were administered at concentrations of 40 and 80 μg/mL concurrently with H_2_O_2_.

### Flow cytometry analysis

Cell apoptosis was assessed using Annexin V-FITC and propidium iodide (PI) staining. After washing with PBS, cells were resuspended to a density of 1 × 10⁶/mL. A 1 mL aliquot (approximately 1 × 10⁵ cells) was transferred into a 5 mL flow cytometry tube and stained with Annexin V-FITC (BD Pharmingen, USA) and PI. Following a 15-minute incubation in the dark at room temperature, apoptotic cells were analyzed using a NovoCyte Quanteon flow cytometer (Agilent, California, USA).

### Assessment of reactive oxygen species and mitochondrial membrane potential

Intracellular reactive oxygen species (ROS) levels were measured using the oxidative stress-sensitive fluorescent probe CM-H_2_DCFDA (Beyotime, Shanghai, China). Caco-2 cells were incubated with 10 μmol/L CM-H_2_DCFDA at 37°C for 30 minutes. After incubation, cells were rinsed twice with PBS and examined under a fluorescence microscope.

The mitochondrial membrane potential (MMP) was evaluated using the JC-1 assay kit (Beyotime). Cells were washed twice with PBS, incubated with JC-1 staining solution at 37°C in the dark for 30 minutes, and then washed with JC-1 buffer. Fluorescence was detected using an Olympus EX71 microscope, and MMP was quantified by the ratio of red (590 nm) to green (520 nm) fluorescence.

### Western blotting

Total protein from colon tissues or Caco-2 cells was extracted and separated via SDS-PAGE, followed by electrotransfer onto PVDF membranes. The membranes were then blocked using QuickBlock Blocking Buffer (Beyotime) and incubated overnight at 4°C with the following primary antibodies: anti-Occludin (1:1,000; lot #GR3243495-14; Abcam, Cambridge, UK), anti-bax (1:1,000; lot #2; Cell Signaling Technology [CST], Danvers, MA, USA), anti-Caspase-3 (1:1,000; lot #19; CST), anti-cleaved-Caspase-3 (1:1,000; lot #19; CST), and anti-GAPDH (1:3,000; lot #8; CST), all sourced from Cell Signaling Technology (Boston, USA). After thorough washing with TBS-T, membranes were exposed to HRP-labeled secondary antibodies (1:3,000; CST) for subsequent detection. Protein bands were visualized using an enhanced chemiluminescence substrate (Millipore, Massachusetts, USA), and images were captured using the Bio-Rad ChemiDoc XRS+ imaging system (Hercules, CA, USA).

### Statistical analysis

All statistical analyses were performed using SPSS software (version 22.0; SPSS Inc., Chicago, IL, USA). Normality of the data were assessed prior to further analysis. Data conforming to a normal distribution were presented as mean ± standard deviation (*x̄* ± *s*). For comparisons among multiple independent groups with normally distributed data, one-way analysis of variance was employed. When the assumption of homogeneity of variance was satisfied, intergroup differences were evaluated using Tukey’s post hoc test. In cases of unequal variances, Dunnett’s T3 test was applied instead. A *P* value of <0.05 was considered statistically significant (*), while *P* < 0.01 denoted a highly significant difference (**).

## RESULTS

### Efficient synthesis of thiolated chitosan/sodium alginate nanoparticles (TCS/ALG/Emodin-NPs) and Emodin encapsulation

**Fig 1:**
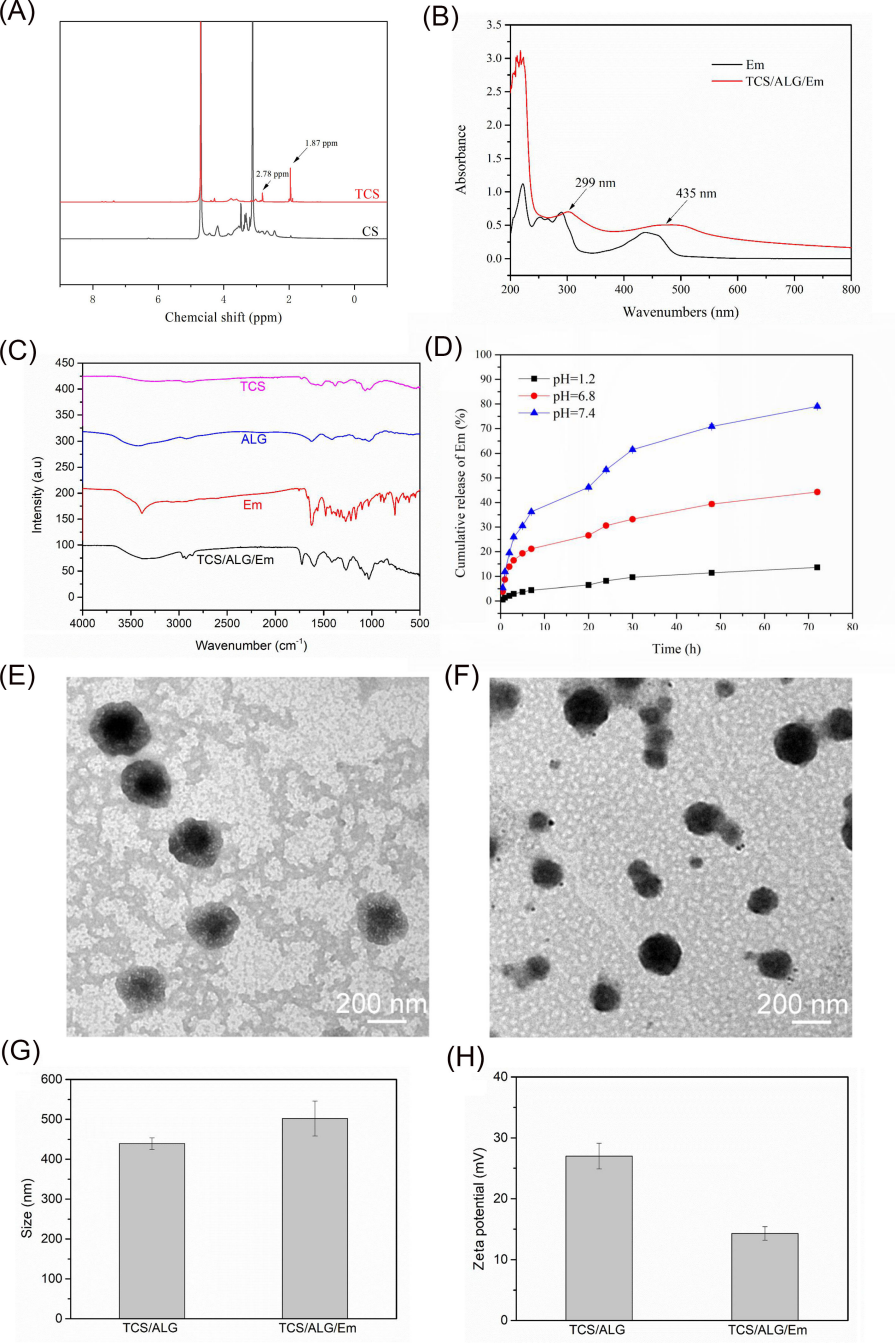
Characterization of TCS/ALG/Emodin-NPs. (**A**) 1^H^ NMR spectrum with characteristic proton peaks of chitosan (CS) and thiolated chitosan (TCS). (**B**) UV–Vis absorption spectra of free Emodin (Em) and TCS/ALG/Emodin-NPs. (**C**) Fourier-transform infrared spectra of TCS, sodium alginate, Em, and TCS/ALG/Emodin-NPs. (**D**) *In vitro* release of Emodin from TCS/ALG/Emodin-NPs under pH 1.2, pH 6.8, and pH 7.4 conditions. (**E**) Morphology of TCS/ALG-NPs determined by electron microscope. (**F**) Morphology of TCS/ALG/Emodin-NPs determined by electron microscope. (**G**) Size distribution spectrum by dynamic light scattering. (**H**) Zeta potential of TCS/ALG/Emodin-NPs determined by laser diffraction.

Figure 1A displays the 1^H^ NMR spectra of CS and thiolated chitosan (TCS). In the TCS spectrum, the N-acetyl methyl proton appears at 1.87 ppm , and a peak at 2.78 ppm corresponds to the methylene group (CH₂–SH) from the thiolated side chain. These spectral features confirm the successful thiolation of chitosan.

Figure 1B illustrates the UV–Vis absorption spectra of free Emodin and TCS/ALG/Emodin-NPs. The TCS/ALG/Emodin-NPs share absorption peaks at 299 and 435 nm with free Emodin, indicating that Emodin was effectively incorporated into the nanoparticle system.

FTIR spectra of TCS, ALG, Em, and TCS/ALG/Emodin-NPs are shown in Fig. 1C. The presence of characteristic peaks from all components in the composite spectrum, along with the disappearance of the Emodin carbonyl band at 1,624 cm⁻¹, further confirms successful nanoparticle formation and drug loading.

TEM images (Fig. 1E and F) show that both TCS/ALG-NPs and TCS/ALG/Emodin-NPs are uniformly distributed, without aggregation, and display near-spherical morphology.

Dynamic light scattering results, presented in Fig. 1G, show that TCS/ALG-NPs have an average diameter of 502 nm with a PDI of 0.53, while TCS/ALG/Emodin-NPs are slightly smaller at 439 nm and display improved homogeneity with a PDI of 0.33. Zeta potential measurements (Fig. 1H) show a surface charge reduction from +27 mV for the TCS/ALG-NPs to +14.3 mV after Emodin loading, indicating successful drug incorporation.

The pH-responsive release profile of Emodin from TCS/ALG/Emodin-NPs was assessed via the dialysis bag method under simulated gastric (pH 1.2), intestinal (pH 6.8), and colonic (pH 7.4) conditions. As shown in Fig. 1D, minimal drug release occurred under pH 1.2 and pH 6.8, whereas a marked release was observed at pH 7.4, demonstrating that the TCS/ALG/Em nanoparticle system possesses favorable colon-targeting properties.

### TCS/ALG/Em nanoparticles (Emodin-NPs) enhance renal function in adenine-induced CKD mice

**Fig 2:**
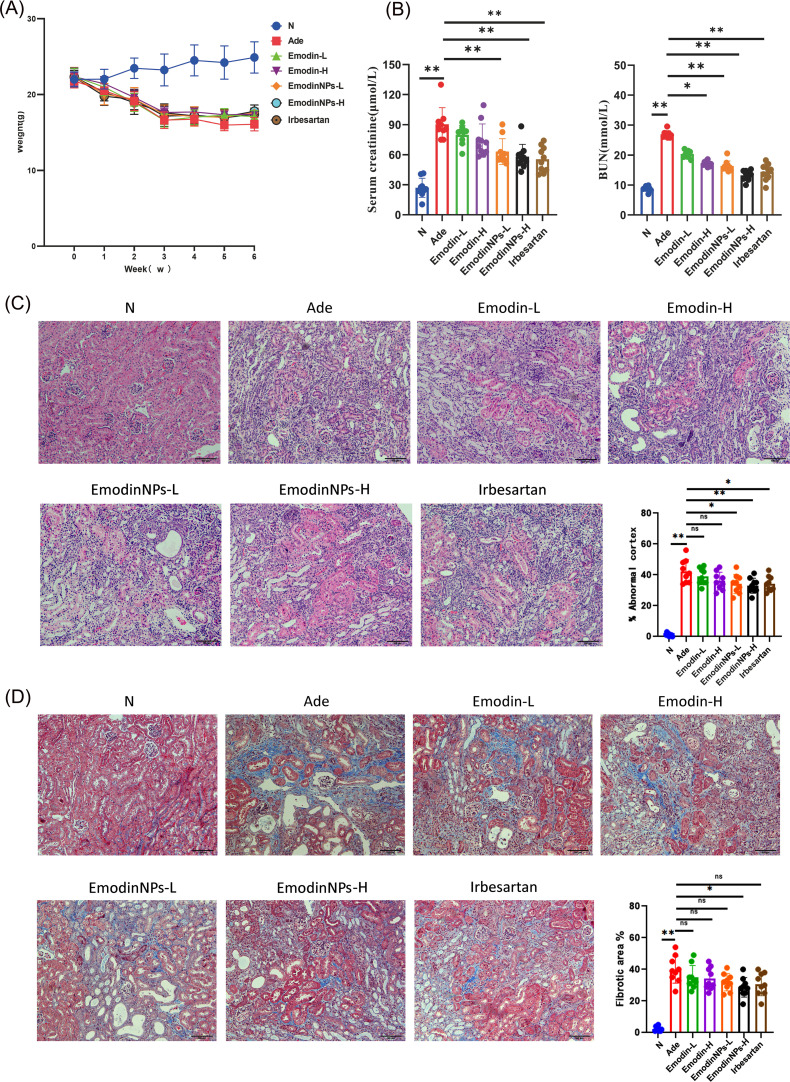
Effects of TCS/ALG/Emodin-NPs on adenine-induced CKD mice. (**A**) Body weight. (**B**) Serum creatinine and urea nitrogen. (**C**) H&E staining of kidney tissue (100×) and the extent of abnormal renal parenchyma by H&E staining. (**D**) Masson’s trichrome staining of kidney tissue (100×) and quantification of kidney fibrosis area. The data are presented as the mean ± standard deviation; *n* = 10 per group. *P < 0.05, **P < 0.01; ns, no statistically significant difference.

Mice administered a 0.2% adenine-containing diet exhibited significant reductions in body weight starting from the first week, with these changes persisting throughout the 6-week period (Fig. 2A), as compared to the normal group. Mice treated with low and high doses of Emodin-NPs showed a mild increase in body weight relative to the untreated Ade group (Fig. 2A). Scr and BUN levels were markedly elevated in the Ade group compared to the normal group (*P* < 0.01, respectively; Fig. 2B). Compared with untreated Ade mice, the levels of Scr and BUN in Ade mice treated with high-dose Emodin-NPs were significantly reduced (*P*<0.01, respectively; Fig. 2B). The therapeutic effect of the Emodin-NPs treatment group was superior to that of the Emodin group (Fig. 2B). The improvement effect of high-dose Emodin-NPs on renal function was comparable to that of the positive drug irbesartan (Fig. 2B). The renoprotective effect was more pronounced with the high dose of Emodin-NPs compared to the low dose (Fig. 2B).

### Emodin-NPs mitigate renal injury in adenine-induced mice

To evaluate the protective effects of Emodin-NPs on renal pathology, we conducted H&E and Masson’s trichrome staining on kidney paraffin sections. Normal group mice exhibited intact renal tubular structures without signs of interstitial damage and fibrosis (Fig. 2C and D). In contrast, untreated Ade mice displayed marked renal tubule atrophy, tubular dilation, mononuclear cell infiltration, and prominent interstitial fibrosis (Fig. 2C and D). Quantitative analysis revealed a significantly higher proportion of abnormal renal parenchyma and fibrotic area in the Ade group compared to the normal group (*P* < 0.01, respectively; Fig. 2C and D). After treatment with high-dose Emodin-NPs, the pathological changes such as tubular atrophy, lumen dilation, interstitial inflammation, and fibrosis in Ade mice were significantly alleviated (*P* < 0.01 and *P* < 0.05, Fig. 2C and D). The therapeutic effect of high-dose Emodin-NPs is superior to that of low-dose Emodin-NPs, and the therapeutic effect of the Emodin-NPs group is better than that of the Emodin group (Fig. 2C and D). Oral administration of Emodin-NPs can mitigate the progression of kidney injury in Ade mice, supporting the potential of Emodin-NPs in slowing the progression of kidney injury in CKD models.

### Oral Emodin-NP treatment enhances intestinal barrier integrity and attenuates inflammation in CKD mice

**Fig 3:**
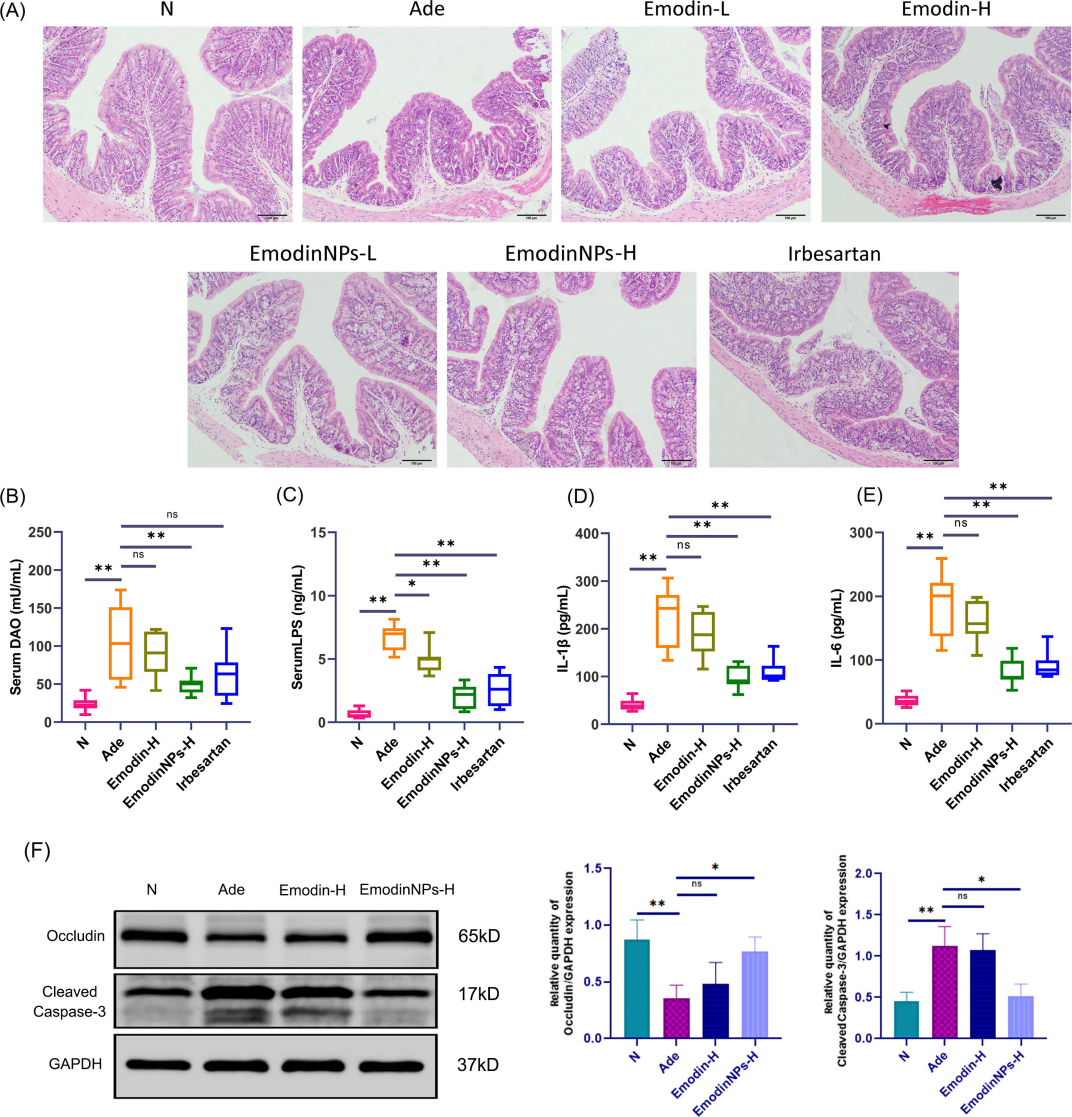
TCS/ALG/Emodin-NPs improved the intestinal barrier function and reduced systemic inflammation in CKD mice. (**A**) H&E staining of colon tissue (100×). (**B–E**) Serum levels of DAO, LPS, IL-1β, and IL-6 by enzyme-linked immunosorbent assay; *n* = 8. Data are presented as the mean ± standard deviation. *P < 0.05, **P < 0.01. (**F**) Western blot analysis of Occludin, cleaved-Caspase-3, and GAPDH in colon tissues. The data are presented as the mean ± standard deviation of the mean for each measurement*; n* = 6. *P < 0.05, **P < 0.01; ns, no statistically significant difference.

Histological analysis using H&E staining of colonic sections from adenine-induced CKD mice revealed pronounced pathological changes, including a reduction in goblet cell numbers, villus structural damage, thinning of the mucus layer, widened epithelial junctions, and notable infiltration of lymphocytes and monocytes (Fig. 3A). Furthermore, serum levels of DAO and LPS were significantly elevated in Ade mice relative to normal group (*P* < 0.01, respectively; Fig. 3B and C), indicating disruption of intestinal barrier function in this model.

Administration of Emodin-NPs via oral gavage notably mitigated mucosal injury in the colon, reduced epithelial damage and inflammatory cell infiltration, and restored mucus layer thickness (Fig. 3A). High-dose Emodin-NPs treatment led to a significant reduction in serum DAO and LPS concentrations (*P* < 0.01, respectively; Fig. 3B and C). The effects of Emodin-NPs surpassed those observed with free Emodin, with the high-dose Emodin-NPs group showing more pronounced protection of the intestinal epithelium and lower circulating levels of DAO, IS, and LPS. Remarkably, the efficacy of high-dose Emodin-NPs in restoring barrier integrity exceeded that of irbesartan, which predominantly targets renal function. These findings suggest that the therapeutic benefits of Emodin-NPs in CKD may be largely attributed to their ability to repair intestinal barrier damage.

Protein expression in colonic tissues, assessed by Western blot, revealed that adenine-induced CKD mice showed significantly reduced levels of the tight junction protein Occludin and elevated expression of Cleaved Caspase-3 when compared to the normal group (*P* < 0.01, respectively; Fig. 3F). Notably, treatment with high-dose Emodin-NPs restored Occludin expression and suppressed cleaved-Caspase-3 levels (*P* < 0.05, respectively; Fig. 3F and G), suggesting that Emodin-NPs can reinforce gut barrier integrity and mitigate epithelial damage.

Additional assessment of inflammatory markers revealed that adenine-induced CKD mice had significantly elevated serum levels of IL-1β and IL-6 compared to controls (*P* < 0.05, respectively; Fig. 3D and E). High-dose Emodin-NPs treatment markedly suppressed these pro-inflammatory cytokines (*P* < 0.05, respectively; Fig. 3D and E), reinforcing the concept that restoring intestinal barrier function is a critical mechanism by which Emodin-NPs alleviate systemic inflammation associated with CKD.

### Modulatory effects of Emodin-NPs on gut microbiota in CKD mice

**Fig 4:**
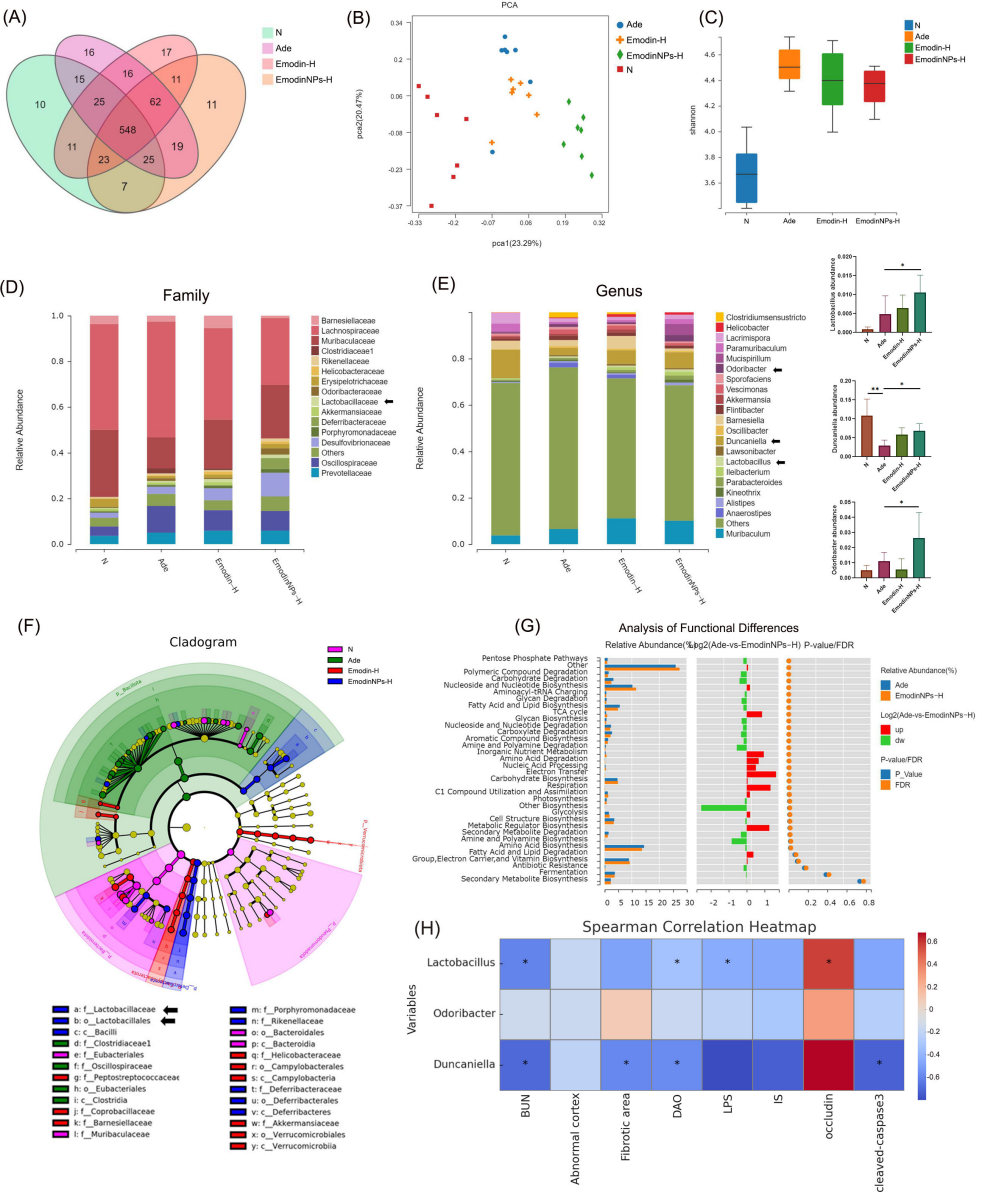
Regulation TCS/ALG/Emodin-NPs on gut microbiome in CKD mice. (**A**) Venn diagram of OTUs. (**B**) Principal component analysis (PCA). (**C**) The Shannon analysis of α-diversity. (**D**) Compositions of gut microbiota at the family level. (**E**) Compositions of gut microbiota at the genus level and analysis of *Lactobacillus*, *Odoribacter*, and *Duncaniella*. (**F**) LEfSe analysis of bacteria with significant effects among the three groups. (**G**) Metacyc function analysis between the Ade group and the Emodin-NPs-H group by PICRUSt2. (**H**) Relationship between gut microbiota and CKD-related indicators by Spearman correlation analysis. The values range from −1 to 1, with colors representing the strength of the correlation, where red indicates a strong positive correlation and blue indicates a strong negative correlation. *P < 0.05,**P < 0.01.

Gut microbiota is a known contributor to compromised intestinal barrier function. To investigate this, 16S rRNA sequencing was conducted on fecal samples from four groups: normal, adenine-induced CKD (Ade), Emodin-H, and high-dose Emodin-NPs (Emodin-NPs-H). Both Venn diagram and principal component analysis demonstrated clear distinctions in OTUs between the normal and Ade groups (Fig. 4A and B). In terms of α-diversity, the Ade group exhibited a significantly elevated Shannon index compared to the normal group, suggesting microbial dysbiosis potentially driven by the proliferation of pathogenic bacteria (Fig. 4C). Treatment with either Emodin-H or Emodin-NPs-H partially reduced the Shannon index, indicating a trend toward microbial homeostasis (Fig. 4C).

Group-wise analysis further revealed that high-dose Emodin-NPs effectively modulated the gut microbial structure, notably enhancing the relative abundance of beneficial genera, including *Lactobacillus*, *Odoribacter*, and *Duncaniella* (Ade VS Emodin-NPs-H, *P* < 0.05, respectively; Fig. 4D and E), especially *Lactobacillus* (Lefse analysis, Fig. 4F), which is the most famous gut probiotics.

Metabolic pathway predictions using PICRUSt2 highlighted distinct functional shifts between the Ade group and those treated with high-dose Emodin-NPs. The altered pathways were mainly related to carbohydrate and polysaccharide degradation, C1 compound metabolism, biosynthesis of secondary metabolites, and lipid and fatty acid synthesis (Fig. 4G).

To further explore the potential relationship between gut microbiota and CKD-related indicators, the correlations between the three main bacterial species obtained in this study and renal injury, intestinal barrier function, and inflammation were calculated based on Spearman correlation analysis. As shown in Fig. 4H, the abundant strains of the Emodin-NPs group (*Lactobacillus*, *Odoribacter*, and *Duncaniella*) were negatively correlated with renal injury, LPS, DAO, and positively correlated with Occludin. It indicates that Emodin-NPs significantly alleviates endotoxemia and intestinal barrier function in CKD by reshaping the structure of the gut microbiota.

### Emodin-NPs attenuate apoptosis and oxidative stress in H_2_O_2_-challenged Caco-2 cells

**Fig 5:**
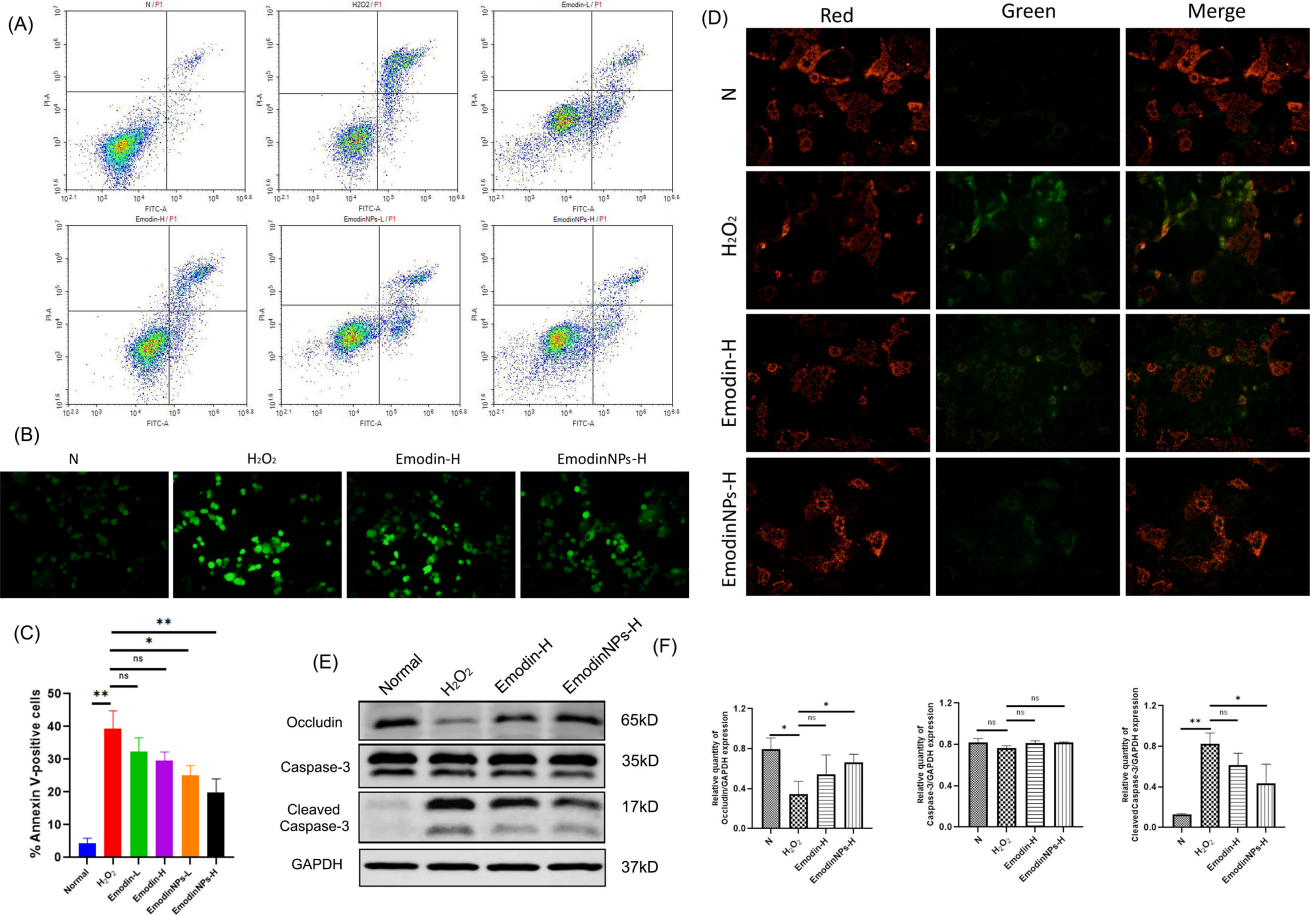
Administration of Emodin-NPs in H_2_O_2_-stimulated Caco-2 cells. (**A**) Annexin/PI assay by flow cytometry. (**B**) ROS analysis. (**C**) Annexin V+ cells analyzed by flow cytometry. (**D**) Mitochondrial membrane potential (JC-1). (**E and F**) Western blot analysis of Occludin, bax, Caspase-3, cleaved-Caspase-3, and GAPDH protein expression in H_2_O_2_-treated and H_2_O_2_ + simultaneous Emodin or Emodin-NPs-treated Caco-2 cells. The data are presented as the mean ± standard deviation; *n* = 3. *P < 0.05, **P < 0.01; ns, no statistically significant difference.

The pro-apoptotic effects of H_2_O_2_ were evaluated using Annexin V/PI staining. After 24 hours of exposure to 100 μM H_2_O_2_, a significant increase in Annexin V-positive Caco-2 cells was observed (*P* < 0.01, Fig. 5A and C), indicating elevated apoptosis. Treatment with Emodin at concentrations of 40 and 80 μg/mL led to a slight reduction in cell apoptosis (Fig. 5A and C). Notably, Emodin nanoparticles (Emodin-NPs) exhibited a more pronounced antiapoptotic effect, especially at higher doses (*P* < 0.05 and *P* < 0.01, Fig. 5A and C).

Intracellular oxidative stress was assessed by quantifying ROS using the H₂DCFDA probe. H_2_O_2_ exposure markedly elevated ROS levels in Caco-2 cells compared with the untreated control group (Fig. 5B). In contrast, co-treatment with 80 μg/mL Emodin-NPs significantly suppressed ROS accumulation in H_2_O_2_-stimulated cells (Fig. 5B).

MMP was evaluated through JC-1 fluorescence microscopy. H_2_O_2_ exposure led to a reduction in MMP, reflected by a shift from red to green fluorescence, indicating mitochondrial impairment (Fig. 5D). However, treatment with high-dose Emodin-NPs (Emodin-NPs-H) preserved mitochondrial integrity, as evidenced by the sustained red fluorescence signal (Fig. 5D).

As illustrated in Fig. 5E and F, H_2_O_2_ treatment notably decreased the expression of Occludin and Bax, while also elevating cleaved-Caspase-3, collectively confirming the induction of apoptotic processes in Caco-2 cells. In contrast, administration of 80 μg/mL Emodin-NPs mitigated these alterations and superior efficacy of the Emodin nanoparticles over the free Emodin (*P* < 0.05, Fig. 5E and F).

In summary, these *in vitro* findings demonstrate that Emodin-NPs effectively mitigate H_2_O_2_-induced oxidative damage and apoptosis in Caco-2 cells, offering direct cytoprotective benefits to colonic epithelial cells.

## DISCUSSION

CKD is a growing global health burden, yet effective disease-modifying treatments remain elusive. Conventional therapies like RAAS and SGLT2 inhibitors slow disease progression but do not reverse renal damage (26–28). In this context, the gut–kidney axis has emerged as a compelling therapeutic target. Gut dysbiosis in CKD contributes to the overproduction of uremic toxins (e.g., IS, PCS, and TMAO), disruption of the intestinal barrier, and systemic inflammation, all of which accelerate renal deterioration (29–31).

This study confirms and extends prior findings that modulation of the gut–kidney axis offers a novel avenue to slow CKD progression. Strategies such as prebiotics, SCFA supplementation, adsorbents (e.g., AST-120), and microbiota-targeted interventions have shown early promise (32–34). However, few have overcome key pharmacological limitations, particularly in the context of delivering microbiota-regulating agents like Emodin with high precision and efficacy.

TCM has shown promising potential in modulating gut microbiota to alleviate CKD, providing a novel therapeutic strategy rooted in both ancient theory and modern microbiome science. Numerous TCM herbs, such as rhubarb and *Astragalus membranaceus*, have been reported to improve renal function by reshaping gut microbiota composition (35, 36). Emodin has demonstrated renoprotective effects via modulation of gut microbiota and inflammatory pathways (37–39). Emodin is known to enhance gut barrier function, reduce systemic endotoxemia, and regulate microbial metabolites such as SCFAs (40, 41). However, its clinical translation is hampered by poor solubility, low oral bioavailability (~3%), and limited colonic targeting (12, 13).

To address these barriers, we developed a thiolated chitosan/sodium alginate nanoparticle system (TCS/ALG/Emodin-NPs) designed for colon-targeted, oral delivery. Chitosan is one of the most extensively studied materials for such delivery systems. Also known as 2-amino-2-deoxy-β-D-glucose, CS is valued for its outstanding biocompatibility. It interacts with colonic mucosal proteins through hydrogen bonding and electrostatic forces, endowing it with considerable bioadhesive strength (42). However, non-covalent interactions alone often fail to sustain drug release at the targeted site, thereby limiting its practical utility. Thiolation of chitosan markedly enhances its adhesive capacity, as the introduction of thiol groups enables the formation of disulfide bonds with the mucus layer and facilitates selective binding to cysteine-rich domains of mucins (43–45).

Sodium alginate, another biocompatible polymer with inherent bioadhesiveness and gel-forming ability, has gained increasing prominence in colon-specific drug delivery. Research demonstrates that alginate-based carriers exhibit greater mucosal adhesion in simulated colonic conditions and degrade more slowly in the absence of microbial enzymes (46, 47). However, in environments enriched with colonic flora and enzymes, the degradation rate is notably accelerated (48). *In vitro* studies show that less than 10% of drug content is released in acidic conditions (pH 1.2), whereas over 90% is liberated under neutral conditions (pH 7.4) containing gut microbiota and enzymes. Incorporating sodium alginate notably improves the drug’s stability in the upper gastrointestinal tract (49). Furthermore, its hydrophilic nature allows for the formation of gels and microspheres, which enhances the encapsulation, stabilization, dispersibility, and solubility of hydrophobic compounds (50, 51). Formulations of Emodin using sodium alginate in nanoparticle or composite carrier form have been shown to significantly improve both solubility and bioavailability.

So, to construct an optimized delivery vehicle, we synthesized thiolated chitosan by coupling the carboxyl group of N-acetylcysteine with the amino group of chitosan through a dehydration condensation reaction. Emodin was then encapsulated using sodium alginate (ALG) and sodium tripolyphosphate as crosslinkers to yield TCS/ALG/Emodin-NPs (Emodin-NPs).

Drug release profiles confirmed that TCS/ALG/Emodin-NPs effectively target the colon via oral administration. In adenine-induced CKD mouse models, treatment with TCS/ALG/Emodin-NPs significantly improved renal function, reduced tubular interstitial injury and fibrosis in a dose-dependent fashion, and demonstrated superior efficacy compared to free Emodin. Microbiome analysis via 16S rRNA sequencing indicated that TCS/ALG/Emodin-NPs alleviated gut dysbiosis, particularly by enhancing the relative abundance of *Lactobacillus*, *Odoribacter*, and *Duncaniella*. Moreover, the Emodin-NPs improved intestinal barrier integrity and reduced serum levels of inflammatory markers such as LPS, IL-1β, and IL-6. *In vitro* assays further revealed that the nanoparticles protected Caco-2 cells from H_2_O_2_-induced apoptosis, restored mitochondrial membrane potential, and lowered ROS levels again, with improved therapeutic outcomes over unformulated Emodin.

One of the key findings is the Emodin-NPs’ ability to modulate the gut microbiota. Through 16S rRNA sequencing, the study revealed that Emodin-NP treatment partially restored gut microbial balance, particularly by increasing the relative abundance of beneficial genera such as *Lactobacillus*, *Odoribacter*, and *Duncaniella*. This is especially meaningful in the context of CKD, where gut dysbiosis contributes to systemic inflammation and disease progression. Restoration of microbial homeostasis is likely a key contributor to the observed decrease in serum levels of endotoxin (LPS) and pro-inflammatory cytokines such as IL-1β and IL-6, indicating a systemic anti-inflammatory effect.

Furthermore, Emodin-NPs strengthened intestinal barrier integrity, which is crucial for preventing the translocation of microbial products that exacerbate kidney inflammation. *In vitro* studies using H_2_O_2_-stimulated Caco-2 cells supported the protective role of Emodin-NPs at the cellular level. The nanoparticles effectively reduced oxidative stress, preserved mitochondrial membrane potential, and decreased apoptosis rates more effectively than free Emodin. These effects underscore the potential of Emodin-NPs to protect intestinal epithelial cells from oxidative injury, which could translate into reduced systemic inflammation and slower CKD progression.

### Conclusion

This study underscores the promise of TCS/ALG/Emodin-NPs as a novel, orally administered therapeutic strategy for CKD. Emodin-NPs offers a comprehensive approach that aligns with the increasingly recognized role of the gut–kidney axis in CKD pathophysiology. Ultimately, this research opens new avenues for the development of microbiota-targeted, anti-inflammatory therapies that can complement existing CKD treatments and potentially slow disease progression in affected patients.

## Data Availability

The 16S rRNA sequencing data have been uploaded to the NCBI Sequence Read Archive (SRA) and the BioProject number is PRJNA1262959.
